# Pelvic plastron secondary to acute appendicitis in a child presented as appendiceal intussusception. A case report

**DOI:** 10.1186/1757-1626-1-135

**Published:** 2008-09-01

**Authors:** Efstratios Christianakis, Anastasios Sakelaropoulos, Constantinos Papantzimas, Michael Pitiakoudis, Georgios Filippou, Dimitrios Filippou, Spiros Rizos, Nikolaos Paschalidis

**Affiliations:** 1 Dept. of Paediatric Surgery, Pendeli Children's Hospital, Palea Pendeli, Athens, Greece; 2 Department of Radiology, Pendeli Children's Hospital, Palea Pendeli, Athens, Greece; 3 First Paediatric Department, Pendeli Children's Hospital, Palea Pendeli, Athens, Greece; 4 Department of Surgery, Medical School, University of Thrace, Alexandroupoli, Greece; 5 Department of Anatomy, Nursing Faculty, University of Athens, Athens, Greece

## Abstract

We report an unusual case of an 11-year-old Greek girl with complicated acute appendicitis. The pelvic plastron that had been formatted secondary to appendix perforation was mimicking appendiceal intussusception in the preoperative ultrasound and computed tomography images. Although acute complicated appendicitis and appendiceal intussusception may represent possible causes of acute abdomen no similar cases have reported in the literature.

## Background

Intussusception of the appendix is a rare type of intussusception with an incidence less than 0.01% [1]. It is usually mimicking acute or chronic abdominal entities and is usually presented as cecal mass. The diagnosis is difficult to be achieved preoperatively. Appendiceal plastron is not an unusual complication of acute appendicitis in children [2]. Appendiceal plastron presented as intussusception has not been presented previously in the literature.

## Case Presentation

An 11-year-old Greek girl presented in the emergency department complaining for intermittent pain in the lower abdomen, as well as for intermittent fever especially during the nights. The symptoms had been presented 14 days ago. The patient had been examined by a doctor who had administrated in an outpatient basis clarithromycin (tabl. 250 mg, 1 × 2, for 5 days) in combination with amoxycillin plus clavulanic acid (tabl. 500 mg, 1 × 3, for three days) because of the possibility of a streptococcal angina infection. At her admission the patient presented lower abdominal pain, fever (T = 37, 5°C) and brownish colour semi diarrhoeic mucous defecations. For six days the patient was treated conservatively, but she did not improve. On the contrary she remained febrile, with diffuse abdominal pain, tenderness and sensibility in press of the right ilium fossa. From the laboratory and imaging exams X-ray chest, urine and stool cultures were normal, and leucosytosis was the only finding WBC: 18,200/μl (Granulocytes: 80, 3%).

**Figure 1:**
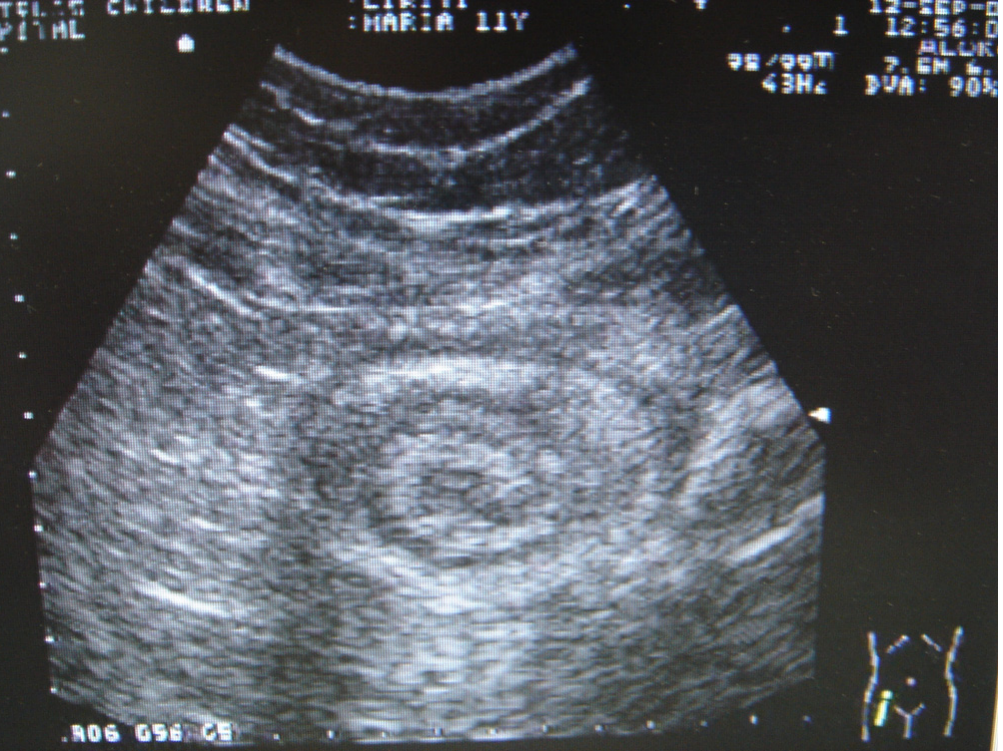
US of the lower right abdomen suggesting intussusception of the appendix. A small quantity of fluid can be noticed.

The ultrasound of the right lower abdominal quadrant demonstrated in transverse incisions a target lesion with multiple hypoechoid and hyperechoid rings. Longitudinal imagination shows heterogeneous tubular mass, suggesting intussusception of the appendix into the cecum, with a small amount of fluid. There was not clear evidence of the appendix. (Figure 1)

**Figure 2:**
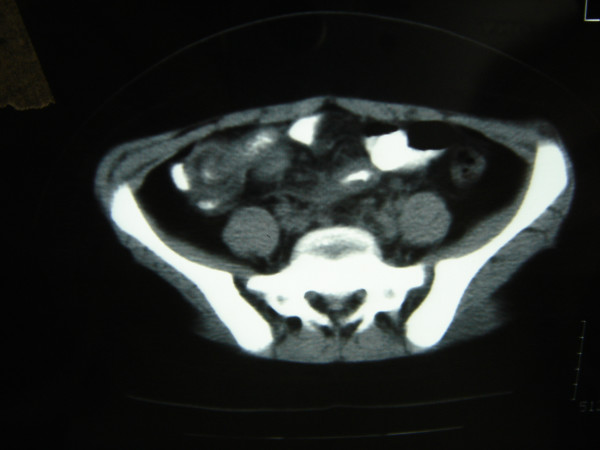
In the CT scan remaining of the contrast liquid in cecum as well as oedema of the cecal wall were observed suggested peri-appendiceal inflammation.

The CT scan with per os administrated contrast suggested cecal intussusception. The main finding was the remaining of the contrast liquid in cecum, the oedema of the cecal wall as well as the misty outline of the mesenteric fat and the lied intestinal loops that suggested peri-appendiceal inflammation. (Figure 2)

The preoperative findings suggested possible appendiceal intussusception and the patient was operated. We used a right sided Pfannenstiel incision. After entering the abdomen an appendiceal pelvic plastron was revealed, while the appendix was not intussuscepted. Because of appendix inflammation, an appendicectomy without burial of the appendix stump was performed. Postoperatively a combination of wide spectrum antibiotics administrated for five days, and the patient discharged at the sixth postoperative day, without presenting any complications.

## Discussion

The most common cause of acute abdomen in children is *acute appendicitis *(AA), which presents diagnostic problems and followed by severe complications. Non-typical symptoms include diarrhoeas similar to those in acute gastroenteritis. Atypical presentation of a pelvic acute appendicitis complicates the paediatric patients with high frequency [3,4].

In our case the initial administration of broad spectrum antibiotics may contribute in the delayed diagnosis. The right abdominal lower quadrant pain on palpation, the low-grade fever (38°C) and the localized tenderness to percussion were the most important clinical signs suggested appendicitis.

*Chronic intussusception (CI) *is another pathological entity of children that is difficult to diagnose. The CI may last more than 14 days and followed by persistent abdominal pain [4-6]. Non-typical presentation may occur in the non-ischemic type of CI which results in a delayed diagnosis. Generally intussusception presents no such typical picture as the acute type of intussusception, with a long history less severe symptoms, like diarrhoea [7,8]. Also there are infrequent attacks of abdominal pain, sporadic vomiting and no, or small changes in defecation [9-11]. Weight loss and abdominal mass assume diagnostic significance [7]. Approximately 3% of all reported acute CI cases occur in infants and about 10% in those over one year of age [11]. The barium enema is usually unsuccessful in hydrostatic reduction and surgery reduction is necessary[6,7,10,11].

## Conclusion

The role of imaging exams (US and CT) in the diagnosis of AA in children may be crucial especially in complicated and obscure cases. In equivocal cases of right lower quadrant pain, these exams may contribute to an earlier diagnosis of AA reducing the rate of unnecessary appendicectomies [12,13]. However, risk groups of children who would benefit most from imaging studies have not been established [14].

## Abbreviations

mg: milligrams; T: temperature; °C: Celcious degrees; WBC: white blood cells; CT: computed tomography; AA: acute appendicitis; CI: chronic intussusception; US: ultrasound

## Competing interests

The authors declare that they have no competing interests.

## Authors' contributions

All authors *(EC, AS, CP, MP, GF, DF, SR, NP) *contributed equally to the patient's therapy, writing the present case report and approving it.

## Consent section

Written informed consent was obtained from the patient for publication of this case report and accompanying images. A copy of the written consent is available for review by the Editor-in-Chief of this journal.
